# Core-Shell Structures of Upconversion Nanocrystals Coated with Silica for Near Infrared Light Enabled Optical Imaging of Cancer Cells

**DOI:** 10.3390/mi9080400

**Published:** 2018-08-14

**Authors:** Kumbam Lingeshwar Reddy, Neeraj Prabhakar, Jessica M. Rosenholm, Venkata Krishnan

**Affiliations:** 1 School of Basic Sciences and Advanced Materials Research Center, Indian Institute of Technology Mandi, Kamand, Mandi 175005, Himachal Pradesh, India; lingeshwarreddykumbam@gmail.com; 2 Pharmaceutical Sciences Laboratory, Faculty of Sciences and Engineering, Åbo Akademi University, 20520 Turku, Finland; nprabhak@abo.fi

**Keywords:** core-shell structures, upconversion nanocrystals, NIR imaging, cancer cells

## Abstract

Optical imaging of cancer cells using near infrared (NIR) light is currently an active area of research, as this spectral region directly corresponds to the therapeutic window of biological tissues. Upconversion nanocrystals are photostable alternatives to conventional fluorophores. In our work, we have prepared upconversion nanocrystals of NaYF_4_:Yb/Er and encapsulated them in silica to form core-shell structures. The as-prepared core-shell nanostructures have been characterized for their structure, morphology, and optical properties using X-ray diffraction, transmission electron microscopy coupled with elemental mapping, and upconversion luminescence spectroscopy, respectively. The cytotoxicity examined using cell viability assay indicated a low level of toxicity of these core-shell nanostructures. Finally, these core-shell nanostructures have been utilized as photostable probes for NIR light enabled optical imaging of human breast cancer cells. This work paves the way for the development of advanced photostable, biocompatible, low-toxic core-shell nanostructures for potential optical imaging of biological cells and tissues.

## 1. Introduction

Optical imaging is a useful technique to visualize the structural and functional evolution of biological materials with negligible invasion, and without disturbing the usual metabolic activities [1,2,3]. Luminescent labels are required for optical imaging of biological materials, since most biomolecules do not have measurable fluorescent signals [4,5,6]. In biomedical research there is an enormous demand for fluorescent probes which emit light at particular wavelengths and can be detected under *in vivo* and *in vitro* conditions through fluorescence microscopy [7,8]. Despite sensitivity and other benefits, traditional fluorescent molecular probes have a number of limitations, such as low penetration depth of excitation light, high toxicity due to the longer exposure needed at shorter wavelengths, auto-fluorescence and strong light scattering from biological tissues that cause low signal to noise ratio (SNR), and photo-bleaching [9,10]. These undesirable properties of conventional organic fluorophores motivate researchers to look for alternatives in luminescent materials, such as quantum dots, carbon-based nanostructures (e.g., nanodiamonds, carbon dots), and upconverting phosphors. For instance, near infrared (NIR) active inorganic luminescent materials can potentially overcome the limitations of traditional fluorescent molecular probes [11].

In recent years, different research groups have prepared NIR active luminescent nanoparticles and efficiently used them for optical imaging; amongst these, considerable attention has been devoted to upconversion luminescent nanomaterials [12,13,14]. Compared to the emitted light, longer wavelength excitation is required for upconversion photoluminescence. Since this process is quite opposite to Stokes-shift, it is also called anti-Stokes shift [15]. Lately, much interest has especially been directed to lanthanide doped upconversion nanocrystals (UCNC) [16]. Though UCNC have low quantum yield, these nanocrystals have numerous outstanding abilities, for instance: Long Stokes-shift, sharp absorption and emission peaks, extended lifetimes, excessive photostability, no interference with the auto-fluorescence of biological tissues, and minimal photo-toxicity towards biological tissues [17,18,19,20,21,22,23]. The abovementioned advantages make UCNC a fascinating material for most of the applications in various fields including biomedicine [24,25,26]. A further advantage of UCNC is that they can be used as constructs for the easy preparation of bioprobes for imaging applications [27,28,29,30].

Synthesis of monodispersed, single crystal, high quality nanocrystals (NC) with pure crystal phase and well-defined shapes is of utmost importance for successful biomedical applications such as bioimaging, drug delivery, photodynamic therapy, biosensing, etc. [19,31,32,33,34,35,36]. Most of the commonly used UCNC synthesis procedures, such as thermal decomposition [37], high temperature co-precipitation [38,39], solvo/hydrothermal [40], co-precipitation [41], sol-gel methods [42], combustion synthesis [43], microemulsion [44], electrospinning [45], flaming synthesis [46], and microwave synthesis [47], produce hydrophobic surfaced nanostructures. Compared to the abovementioned methods, microwave assisted synthesis procedures are relatively quick, eco-friendly, and energy efficient. Few works have been reported on the preparation of UCNC with microwave assisted technique, for example, the ionic liquid based route was introduced by Chen et al. for the preparation of NaYF_4_:Yb/Er UCNC with microwave assistance [48]. Wang et al. used air sensitive precursors (such as trifluoroacetates) for the synthesis of Er, Yb doped NaYF_4_ NC [49]. 

One of the essential requirements to make use of nanoparticles in biological environments is that the nanoparticles should have a hydrophilic surface [50]. Therefore, a huge variety of surface alteration approaches, such as addition of hydrophilic layers on and/or chemical modification of the surface, have been developed to convert hydrophobic surfaces into hydrophilic surfaces [51,52,53,54]. Among the several methods, growth of a silica shell on UCNC has attracted great interest since they exhibit low cytotoxicity and their chemically active surface can be easily modified to introduce various functional groups, which can satisfy the needs of conjugating biological molecules or functional nanoparticles [55]. Despite the progress achieved in this field, there is still scope for improvement with regard to the synthesis strategies employed for the preparation of upconversion nanostructures, such as post-synthesis surface modification strategies to tune the wettability and consequently biocompatibility, stability, and dispersability behavior of the nanoparticles and in examining the uptake of the nanoparticles by cells for optical imaging applications. Keeping these points in mind, in the present work, we have prepared core-shell nanostructures of lanthanide doped upconversion nanocrystals coated with silica. Silica coatings are a commonly applied strategy to enhance the dispersability of nanoparticles in biological or physiological environments, due to the inherent hydrophobicity of most other inorganic nanoparticles [56]. Deposition of a silica coating further endows the core material (in this case the UCNC) with enhanced colloidal, chemical, photo-, and thermal stability, provides controlled porosity and surface chemistry, easy processing of both coating and any further surface functionalizations, and is optically transparent [57]. The as-prepared core-shell nanostructures have been examined for their cytocompatibility, after which they have been used for cellular labeling and finally utilized as photostable probes for NIR light enabled optical imaging of human breast cancer cells. We anticipate that this work will pave the way for the development of several advanced photostable and biocompatible core-shell nanostructures not only for optical imaging, but also for several other technological applications.

## 2. Materials and Methods

### 2.1. Materials

Yttrium trichloride hexahydrate (YCl_3_·6H_2_O), ytterbium trichloride hexahydrate (YbCl_3_·6H_2_O), erbium trichloride hexahydrate (ErCl_3_·6H_2_O), sodium hydroxide (NaOH), ammonium fluoride (NH_4_F), acetic acid, ethanol, dimethyl sulfoxide (DMSO), 4′,6-diamidino-2-phenylindole (DAPI), TritonX-100, tetraethyl orthosilicate (TEOS), (3-aminopropyl)triethoxysilane (APTES), 2-(4-iodophenyl)-3-(4-nitrophenyl)-5-(2,4-disulfophenyl)-2H-tetrazolium, monosodium salt (WST-1), and ammonium hydroxide (NH_4_OH) were procured from Sigma-Aldrich, India. All the chemicals received from the vendor were used as they were, without any extra purification. All the experiments were performed with Milli-Q (18.2 MΩ-cm) water.

### 2.2. Synthesis

#### 2.2.1. NaYF_4_:Yb/Er Nanocrystals

Lanthanide (Yb and Er) doped NaYF_4_ nanocrystals were prepared through a microwave assisted synthesis route as earlier reported by our group [58]. In the typical synthesis procedure, YCl_3_ (0.8 mmol), YbCl_3_ (0.18 mmol) and ErCl_3_ (0.02 mmol) were mixed with a solution mixture of 8 mL of ethanol (EtOH), NaOH (7.5 mmol) dissolved in 2 mL of water, 2 mL acetic acid (AcOH) under constant magnetic stirring at room temperature (RT). Lastly, 2 mL water containing 4 mmol of NH_4_F was added drop wise to the above solution mixture. After 30 min of stirring at room temperature, the entire reaction mixture was moved to a microwave reaction vessel (20 mL) and tightly sealed to heat for 3 h at 150 °C with continuous stirring of 600 rpm. Then the complete system was allowed to cool to 40 °C. Last, of all, the products were rinsed with ethanol and water (7:3 *v*/*v*) mixture three times and collected through centrifugation to eliminate any unreacted leftover precursors. Then the UCNC were dried in vacuum at 60 °C.

#### 2.2.2. UCNC@SiO_2_ Core-Shell Nanostructures

UCNC@SiO_2_ core-shell nanostructures synthesis was carried out as per a previous report with some modifications [59]. In brief, 20 mg of UCNC were properly dispersed in 20 mL ethanol and 2 mL of TritonX-100 was added to the above solution while stirring. Stirring was continued for 30 min, then 0.42 mL of NH_4_OH was added and allowed to stir for another 30 min. Lastly, 0.051 mL of tetraethyl orthosilicate (TEOS) was added and allowed it to stir for 6 h. Then silica coated UCNC core-shell nanostructures were collected through centrifugation. Finally, the obtained core-shell nanostructures were dispersed in ethanol and kept at 4 °C in refrigerator for future use. 

### 2.3. Instrumentation

NaYF_4_:Yb/Er UCNC synthesis was done by using Anton Paar Monowave 300′ microwave synthesizer (New Delhi, India). For the collection of powder X-ray diffraction data, Rigaku Smart Lab 9 KW powder X-ray diffractometer (PXRD, New Delhi, India) was used. The transmission electron microscope (TEM) of FEI Tecnai G2 20 S-twin (New Delhi, India) operating at 200 kV was used to acquire the morphology of the nanostructures. Energy dispersive X-ray analysis (EDAX) and elemental mapping were also performed using the same TEM instrument. For upconversion luminescence (UCL) measurements, 980 nm laser diode (CW, 500 mW, purchased from Optochem International, New Delhi, India) equipped Cary Eclipse fluorescence spectrophotometer was used. For UCL measurements, 1 mg of samples (UCNC and UCNC@SiO_2_) was dispersed in 1 mL of Milli-Q water, followed by 5 min of sonication. Subsequently, the sample was transferred to a 1 mL cuvette and measurements were performed. Leica SP5 STED/MP microscope (New Delhi, India) was used for cell imaging, wherein for excitation, a Ti:sapphire femtosecond pulse laser (Chameleon Ulta, Coherent Inc., Santa Clara, CA, USA) at 980 nm was used. In this case, a standard PMT detector and a 63× oil immersion objective (Leica) was used for imaging the cells. 

### 2.4. Cell Viability Assessment

2-(4-iodophenyl)-3-(4-nitrophenyl)-5-(2,4-disulfophenyl)-2H-tetrazolium monosodium salt (WST-1) cell viability assay was used to measure cell toxicity. MDA-MB-231, Human breast cancer cells, were developed in a 96 well microplate (7000 cells/well) in cell growth medium (DMEM) and then incubated at 37 °C, 5% CO_2_ for 24 h. From each well, the growth medium was replaced with the addition of UCNC and UCNC@SiO_2_, which was prepared in 4-(2-hydroxyethyl)-1-piperazineethanesulfonic acid) (HEPES) buffer (pH 7.4). Next, 5, 10, 25 µg mL^−1^ concentration of particles were added to the cell media and incubated for 48 h. Then 10 µL of WST-1 was added to each well and incubated again for 2 h at 37 °C, 5% CO_2_. After the incubation, the 96 well plate was read by a Varioskan Flash Multimode Reader (Thermo Scientific Inc., Waltham, MA, USA) to get the absorbance at 440 nm. Averaged absorbance readings were plotted and the viable cells were correlated with the absorbance of each concentration of particles.

### 2.5. Cancer Cell Imaging

The MDA-MB-231 cells were plated over glass coverslips using cell media (DMEM) at 5% CO_2_ and 37 °C. Cells (7 × 10^4^ cells mL^−1^) were placed on a fresh coverslip and left to stick for 24 h. Later the cells were cleaned with phosphate buffer solution (PBS) then incubated with UCNC and UCNC@SiO_2_ (5 µg mL^−1^ dispersed in DMEM) for 48 h at 5% CO_2_ and 37 °C. Then 4% paraformaldehyde was used to fix the cells and *cell* sample was mounted using *VECTASHIELD^®^* with *DAPI* for optical microscopy. TCS SP5 MP (Multi-photon, Leica microsystems), HyD detectors, 100× (HCX PL APO CS 100.0×/1.40 OIL) oil objective and LASAF software (Leica application suite) consisting multi-photon microscopy setup was used for imaging. For excitation, Ti-sapphire femtosecond pulse at 980 nm (1.07 W) was used while performing multi-photon microscopy. The green and red emissions were collected in 520–570 nm and 630–680 nm, respectively.

## 3. Results and Discussion

**Scheme 1:**
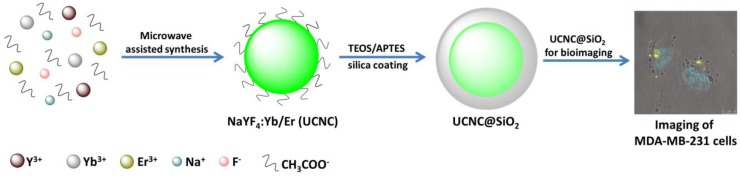
Schematic illustration of upconversion nanocrystals (UCNC)@SiO_2_ synthesis and their application in cancer cell imaging.

The synthesis of UCNC@SiO_2_ and their application in cancer cell imaging is schematically demonstrated in Scheme 1. 

**Figure 1:**
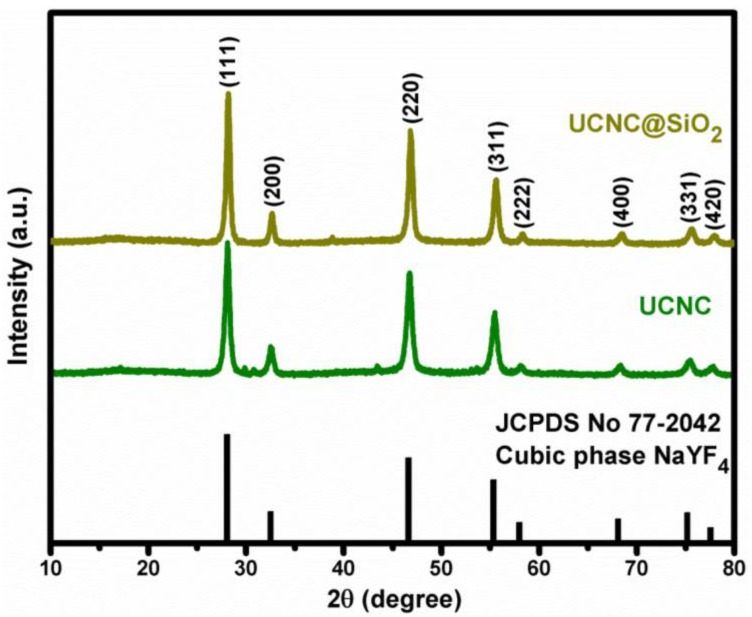
Powder X-ray diffraction pattern of upconversion nanocrystals (UCNC) and UCNC@SiO_2_.

To study structural and phase properties, UCNC and UCNC@SiO_2_ were characterized using X-ray diffraction technique and the obtained patterns are shown in Figure 1. The diffraction peaks displayed at 2θ = 28.1°, 32.5°, 46.8°, 55.5°, 58.1°, 68.4°, 75.5°, and 77.8° could be attributed to (111), (200), (220), (311), (222), (400), (331), and (420) Miller indices, respectively. The XRD pattern of UCNC is exactly matching with prior reported data of cubic phase (JCPDS No. 77-2042) [60]. UCNC@SiO_2_ diffraction peaks were also obtained at the same 2θ position where the core (UCNCs) peaks were evidenced without any additional peaks, indicating that the formation of core-shell nanostructures does not alter the crystallinity of the core UCNC, and also do not lead to the formation of any additional compounds. In accordance with the amorphous nature of the silica shell around UCNC, the peaks corresponding to SiO_2_ could not be evidenced.

**Figure 2:**
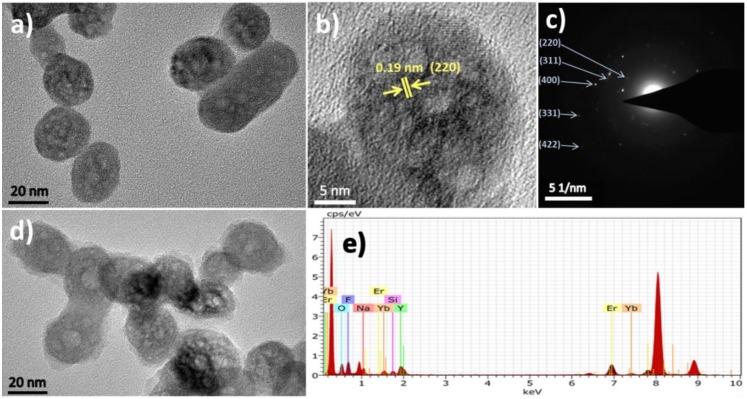
Transmission electron microscope (TEM) image of (**a**) NaYF_4_:Yb/Er UCNC, (**b**) High-resolution TEM image of NaYF_4_:Yb/Er UCNC, (**c**) selected area electron diffraction (SAED) patterns of NaYF_4_:Yb/Er UCNC, (**d**) UCNC@SiO_2_, and (**e**) energy dispersive X-ray analysis (EDAX) of UCNC@SiO_2_.

**Figure 3:**
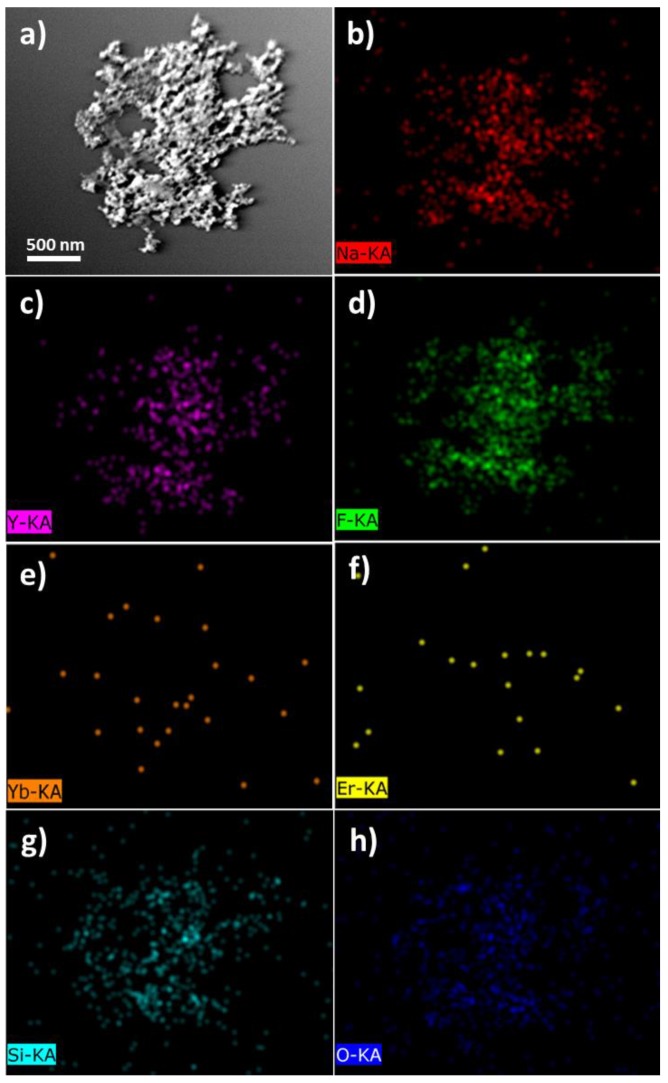
(**a**) STEM-HAADF image of UCNC@SiO_2_ core-shell nanostructures with associated (**b**) sodium (Na), (**c**) yttrium (Y), (**d**) fluoride (F), (**e**) ytterbium (Yb), (**f**) erbium (Er), (**g**) silicon (Si), and (**h**) oxygen (O) elemental mapping.

To investigate the morphology and size of the UCNC and core-shell nanostructures, TEM images were acquired (shown in Figure 2). Figure 2a shows the TEM image of microwave assisted synthesized NaYF_4_:Yb/Er nanocrystals. These particles are of spherical shape with an average size of approximately 25 nm. Figure 2b shows a high-resolution TEM (HRTEM) image of UCNC, which reveals the cubic phase of NaYF_4_ with lattice distance of 0.19 nm, also confirming the *d*-spacing for (220) Miller indices of the material. The selected area electron diffraction (SAED) patterns presented in Figure 2c clearly shows the diffraction rings corresponding to the cubic NaYF_4_ lattice. The thin silica layer coated on the UCNC to form core-shell nanostructures can be clearly seen in Figure 2d. The energy dispersive X-ray analysis (EDAX) of the core-shell nanostructures shown in Figure 2e clearly indicates the existence of all the constituent elements. In addition, the existence of all integral elements such as, sodium (Na), ytterbium (Yb), yttrium (Y), erbium (Er), fluoride (F), silicon (Si), and oxygen (O) was confirmed by TEM elemental mapping of UCNC@SiO_2_ core-shell nanostructures and is presented in Figure 3.

**Figure 4:**
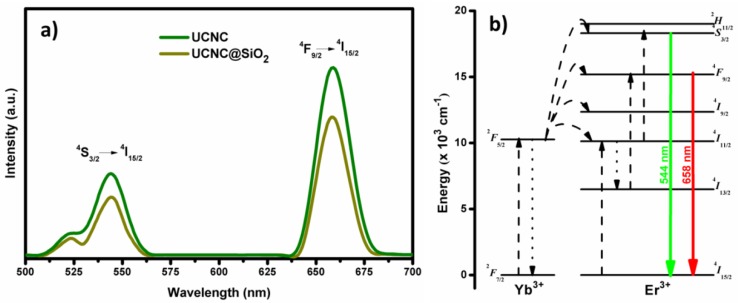
Upconversion luminescence spectra of (**a**) NaYF_4_:Yb/Er and silica-coated excited at 980nm and (**b**) Schematic illustration of lanthanide ions (Yb^3+^, Er^3+^) energy transitions in NaYF_4_:Yb/Er nanocrystals.

Upconversion luminescence (UCL) of UCNC@SiO_2_ core-shell nanostructures was measured at room temperature by excitation at 980 nm wavelength through infrared continuous laser. Two bands have been observed in the UCL spectra of UCNC@SiO_2_ at 658 nm and 544 nm, which is shown in Figure 4a. These upconversion emissions are the results of Er^3+^ ions electronic transitions from ^4^F_9/2_ → ^4^I_15/2_ and ^4^S_3/2_ → ^4^I_15/2_, respectively, and the transitions are schematically shown in Figure 4b [61]. 

**Figure 5:**
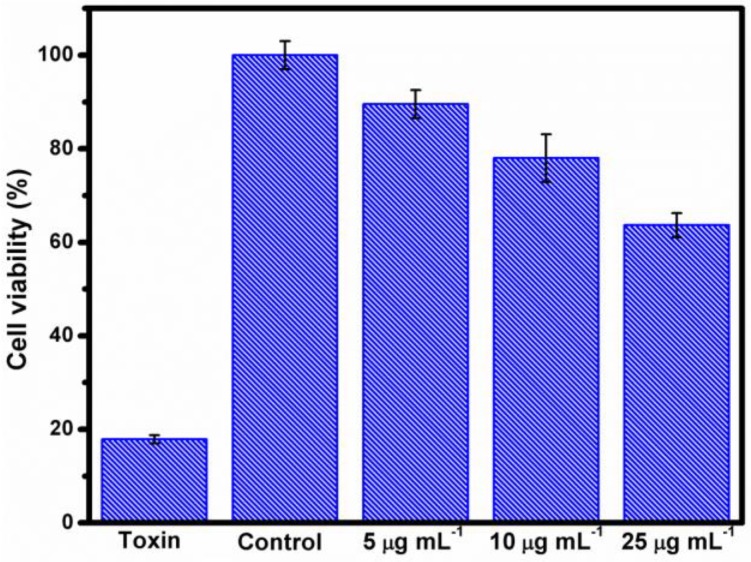
MDA-MB-231 cell viability was assessed by cell proliferation assay (WST-1) on incubation of UCNC@SiO_2_ for 48 h. The cell viability was assessed with positive (DMSO) and negative (media only) controls. The cells incubated with 10% DMSO (toxin), showed efficient toxicity with comparatively lowest cell survival or proliferation. However, the negative control cells have approximately 100% cell proliferation. The UCNC@SiO_2_ treated cells have shown concentration-dependent cell proliferation i.e. the higher the particle concentration, the lower the cell proliferation. The measured cell viability for 5 μg mL^−1^, 10 μg mL^−1^, and 25 μg mL^−1^ UCNC@SiO_2_ treated cells were approximately 90%, 80%, and 70%, respectively.

Before performing cell imaging studies, cytotoxicity experiments were performed using a cell proliferation assay (WST-1). The cell toxicity induced by nanoparticulate imaging probes is mainly associated with their concentration and their surface functionalization [62,63]. In our cell viability experiment, DMSO was used as a positive control, as it is a known cell growth inhibitor and thus behaves like an effective toxin. Therefore, DMSO (10%) was mixed with the DMEM (cell growth media) as a toxin (positive control), while as a negative control, untreated cells (merely DMEM) were used. The untreated UCNC@SiO_2_ core-shell nanostructures were measured in variable concentrations up to 25 μg mL^−1^. The cell proliferation assay was used to evaluate the effect of UCNC@SiO_2_ core-shell nanostructures on the proliferation of human breast cancer cells (MDA-MB-231) after 48 h. The cell viability data is shown in Figure 5. The cell proliferation with UCNC@SiO_2_ did not induce significant toxicity at lower concentrations, and the proliferation of untreated cells is comparable. Nevertheless, a reduction in cell proliferation was noticed with the regular augmentation of UCNC@SiO_2_ nanostructure concentrations in cell growth media.

**Figure 6:**
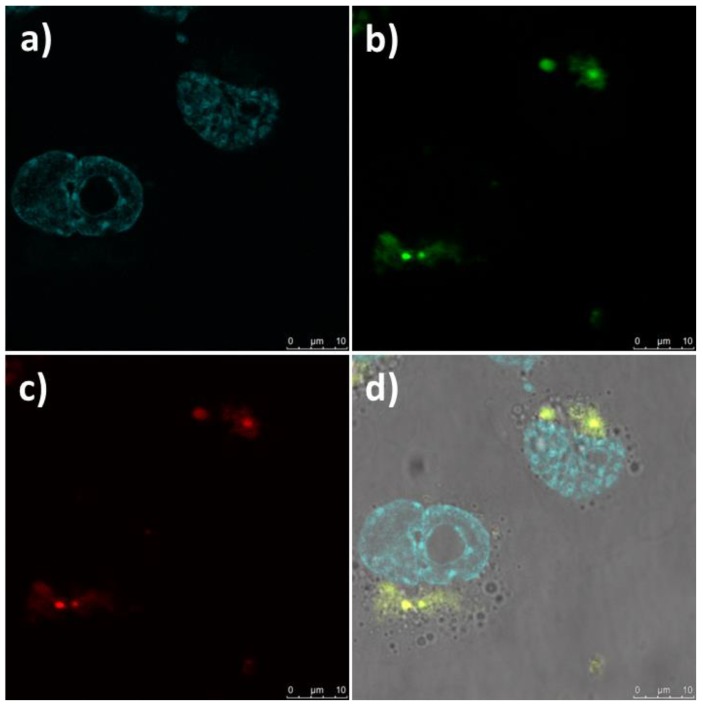
The imaging potential of developed UCNC@SiO_2_ was demonstrated by performing *in vitro* imaging of MDA-MB-231 cells by multi-photon excitation (980 nm laser excitation). The developed UCNC@SiO_2_ has bright, dual color emission in both green and red channels. The UCNC@SiO_2_ imaging further demonstrated that particles are localized in the cytoplasmic space after uptake. (**a**) Cell nuclei were visualized by 4′,6-diamidino-2-phenylindole (DAPI) staining, (**b**) The UCNC@SiO_2_ can be seen as localized spots inside cells. The green emission form UCNC@SiO_2_ was detected in 520–570 nm range. (**c**) The red emission from UCNC@SiO_2_ can be seen again as localized spots in cells. The red emission from UCNC@SiO_2_ was detected in 630–680 nm range. (**d**) Overlay image of (**a**–**c**) with bright-field.

Based on optical properties and cell toxicity investigations of UCNC@SiO_2_, *in vitro* imaging of human breast cancer cells was performed by incubating them with 10 μg mL^−1^ UCNC@SiO_2_ core-shell nanostructures for 48 h, and the resulting images are presented in Figure 6. To investigate the intracellular location of UCNC in the cancer cells, their nucleus was stained with DAPI (Figure 6a). It can clearly be seen (Figure 6b,c) that a cluster of cells displayed bright signal in both the green and red channels, upon an excitation with 980 nm multi-photon excitation, which demonstrates that the UCNC@SiO_2_ core-shell nanostructures are capable of internalization into the human breast cancer cells. UCL emission of core-shell nanostructures was mainly localized in the cytoplasm of the cells, and we did not observe luminescence in the nucleus. The overlay image is presented in Figure 6d. The UCNC@SiO_2_ core-shell nanostructures showed sufficiently good cell viability and efficacy of cellular labeling for optical imaging applications even without further surface functionalization, which could be readily applied on to the silica coating for promoting further cellular uptake or other functions [64]. Importantly, auto-fluorescence of MDA-MB-231 cells can be removed by excitation at longer wavelengths, i.e., 980 nm laser. Consequently, UCNC@SiO_2_ is capable of multi-photon excitation with longer wavelengths, which, in turn, allows less scattering, deep penetration, low tissue absorption, and low autofluorescence for imaging of biological samples [65,66]. 

## 4. Conclusions

In summary, we have synthesized NaYF_4_:Yb/Er upconversion nanocrystals and coated them with silica to procedure core-shell nanostructures. Powder X-ray diffraction pattern of UCNC@SiO_2_ core-shell structures confirms that the core UCNC are in cubic phase. TEM images show that the core-shell nanostructures are predominantly spherical in shape comprising a thin silica shell around the UCNC core of about 25 nm size. The presence of all the constituent elements was confirmed through EDAX and elemental mapping studies. Upconversion photoluminescence measurements proved that the UCNC@SiO_2_ core-shell nanostructures display very good upconversion luminescence. Cell viability experiments through MTT assay shows low cytotoxicity of the nanostructure. Finally, we have demonstrated the bioimaging capability of the UCNC@SiO_2_ core-shell nanostructures through NIR light enabled optical imaging of breast cancer cells. This work opens the way for the development of low-toxic photostable alternatives to traditional fluorophores for bioimaging applications.
